# Chemotherapy-Related Adverse Drug Reaction (ADR) Profile in Geriatric Cancer Patients: A Prospective Study From Western India

**DOI:** 10.7759/cureus.106762

**Published:** 2026-04-10

**Authors:** Priyanka Srivastava, Nitiraj Shete, Barna Ganguly, Nirav N Asarawala

**Affiliations:** 1 Medical Oncology, Rayos Comprehensive Cancer Care, Anand, IND; 2 Biostatistics, Muljibhai Patel Urological Hospital, Nadiad, IND; 3 Pharmacology, Pramukhswami Medical College, Anand, IND; 4 Medical Oncology, M.S. Patel Cancer Centre, Bhaikaka University, Anand, IND

**Keywords:** adverse drug reactions, chemotherapy dose, geriatric assessment, geriatric oncology, western indian population

## Abstract

Background

Chemotherapy adverse drug reactions (ADRs) among geriatric cancer patients (≥65 years) are a major cause of morbidity. Real-world data for the Western Indian population are lacking. In the absence of data and guidelines, geriatric patients either suffer treatment-related toxicities or remain undertreated. The present study aimed to study the clinical profile, geriatric assessment (GA), chemotherapy-related ADRs, and survival outcomes.

Methods

This prospective, observational study enrolled patients aged ≥65 years who were starting curative-intent chemotherapy. Baseline clinical and GA was performed. Treatment details, including planned chemotherapy regimens and modifications in chemotherapy dosing, schedule, or protocol selection based on initial assessments, were recorded. Chemotherapy-related ADRs were assessed using the Common Terminology Criteria for Adverse Events version 5.0 (CTCAE v5.0). Grade 3-5 adverse events were defined as severe. In total, 150 patients were accrued. Patients were followed up for one year post-chemotherapy.

Results

Out of 150 patients, 72 patients (48%) completed all planned cycles. Forty-nine patients (32.66%) reported severe-grade ADRs (CTCAE grades 3, 4, and 5). A total of nine (6%) deaths were reported during chemotherapy (i.e., CTCAE grade 5). The baseline GA parameters were not associated with CTCAE grade (p value > 0.05, nonsignificant). The estimated Kaplan-Meier survival at the end of four years of follow-up suggests worse outcomes in patients who experienced severe-grade ADRs and were unable to complete all the planned chemotherapy cycles.

Conclusions

A significant proportion of geriatric cancer patients experience severe-grade chemotherapy ADRs. Completing planned chemotherapy cycles in patients being treated with curative intent is an important determinant of survival. Patients who experience severe-grade toxicities have adverse survival outcomes, emphasizing the need for personalized toxicity management.

## Introduction

An adverse drug reaction (ADR) is defined as a response to a drug that is noxious and unintended and that occurs at doses normally used for prophylaxis, diagnosis, or therapy of disease or the modification of physiologic function [1]. Chemotherapy drugs are associated with significant ADRs. The incidence of cancer is increasing worldwide, disproportionately in the geriatric age group (≥65 years). Physiological changes associated with aging alter pharmacokinetics (drug handling) and pharmacodynamics (drug effects), often leading to higher and more prolonged drug concentrations, increased sensitivity, and ADRs to medications [2]. Aging affects all four pharmacokinetic steps, including drug absorption, distribution, metabolism, and elimination. Altered receptor sensitivity, slowed drug metabolism, alterations in homeostatic mechanisms, coexisting comorbidities, and polypharmacy are important pharmacodynamic (PD) changes seen in geriatric patients, leading to greater vulnerability [3].

At the same time, there is a lack of evidence regarding medication efficacy and safety for older adults, as geriatric patients are significantly underrepresented in clinical trials. The key barriers include restrictive eligibility criteria, fear of adverse effects in frail populations, comorbidities, logistical challenges, cognitive impairment, and the potential for higher mortality rates from other causes. As a result, there is a significant lack of information on the safety and efficacy of cancer treatment for the growing number of geriatric cancer patients. This leads to either extrapolating data from relatively healthier younger adults, resulting in exaggerated ADRs, or underdosing geriatric cancer patients.

This care gap is recognized worldwide. It has been highlighted in various collaborative group meetings; for example, the Cancer and Aging Research Group, in collaboration with the National Cancer Institute, the National Institute on Aging, and the Alliance for Clinical Trials in Oncology, held a conference in November 2012. It was also recognized that geriatric assessment (GA), rather than chronological age, is a better tool for assessing physiological age and can identify older geriatric patients at higher risk of cancer treatment-related toxicities [4]. The American Society of Clinical Oncology recommends that all geriatric oncology patients undergo a comprehensive GA (CGA) of various domains, including social support, physical function/falls, cognition, nutrition, medications, comorbid medical disorders, and depression [5].

There is a lack of real-world data on chemotherapy ADRs in Indian geriatric cancer patients. CGA is time-consuming, and there are limitations in performing complete assessments in resource-limited settings. Prospective studies evaluating the performance of GA in predicting ADRs and survival outcomes in the Indian population are lacking, and clinicians generally need to extrapolate results from studies conducted in other populations [6]. To address this knowledge gap, the present prospective study aimed to study the clinical profile, chemotherapy-related ADRs, and survival outcomes in geriatric cancer patients from Western India.

## Materials and methods

This prospective, observational study was conducted at a university cancer hospital and research center in western India. This study is a part of a PhD study dealing with chemotherapy ADRs in geriatric cancer patients treated with curative intent.

The primary objective of this study was to study ADRs related to chemotherapy in geriatric oncology patients treated with curative intent. Secondary objectives were to study the clinical profile including geriatric assessment, chemotherapy prescription practices, and survival outcomes.

Institutional Ethics Committee approval was obtained in April 2021, and patients were enrolled between April 2021 and March 2023. Patients were followed up for a period of one year post-chemotherapy. Written informed consent was obtained from all participants before enrollment. The study adhered to ethical guidelines established by the Declaration of Helsinki, Good Clinical Practice guidelines, and the Indian Council of Medical Research regulations.

The study included geriatric cancer patients aged 65 years and above who were undergoing chemotherapy with curative intent. Patients were eligible if they had a histologically confirmed malignancy, an Eastern Cooperative Oncology Group (ECOG) performance status of ≤2 [7], and were scheduled to receive curative-intent chemotherapy. Exclusion criteria included patients receiving palliative chemotherapy, those with cognitive impairment, and those unwilling or unable to provide informed consent.

Patient data were collected systematically using structured case record forms. Baseline demographic information, including age, gender, and socioeconomic status (assessed using the Modified Kuppuswamy Socioeconomic Scale) [8], was recorded. Clinical examination findings, such as presenting symptoms, disease duration, type of cancer diagnosis, stage, ECOG performance status, nutritional status, and BMI [9], were also documented. Laboratory and radiological investigations were reviewed to establish the clinicopathological profile of each patient. Treatment details, including planned surgery, radiation therapy, and chemotherapy regimens, were noted, along with pre-chemotherapy blood investigations and any modifications in chemotherapy dosing, scheduling, or protocol selection based on initial assessments.

GA included assessment of mood and depressive symptoms using the Geriatric Depression Scale-4 (GDS-4) [10] and social functioning and support using the Medical Outcomes Study Social Support Survey (MOS-SSS) [11]. Chemotherapy-related ADRs were assessed using the Common Terminology Criteria for Adverse Events version 5.0 (CTCAE v5.0) [12]. Overall survival (OS) was defined as the time from diagnosis to death or last follow-up, while relapse-free survival was defined as the time from diagnosis to disease recurrence, primary progression, or death. GDS-4, Modified Kuppuswamy Socioeconomic Scale, MOS-SSS forms, and CTCAE v5.0 are open-access tools available in the public domain. Patients were followed up for one year post-chemotherapy. Final patient enrollment was completed in 2023; patients who completed chemotherapy were followed up until December 2024. Survival data capture for all patients was performed until study completion in December 2024.

The sample size was calculated based on two primary outcomes of interest: chemotherapy-related ADRs and mortality. Literature review suggested an estimated chemotherapy-related ADR rate of approximately 30% in older adults globally, while a pre-assumed ADR rate of 44% was considered for cancer patients in the study setting. To detect this difference with 80% power at a 5% level of significance, a minimum of 99 patients was required. Similarly, for mortality assessment, an estimated global mortality rate of 54% in older adults was compared to a pre-assumed local mortality rate of 40%, requiring a minimum of 99 patients for statistical significance. Given that the study was conducted during the COVID-19 pandemic, a higher attrition rate (approximately 50%) was anticipated, leading to a planned enrollment of at least 150 patients to ensure sufficient data for analysis. After screening 164 patients, 150 eligible patients were enrolled in the study. At the end of the study in December 2024, OS data for 125 patients were available; 25 patients were lost to follow-up.

Quantitative variables were summarized as mean ± SD for normally distributed data and median with IQR for nonnormally distributed data. Qualitative variables were presented as frequencies and percentages. The chi-square test was used to assess associations between categorical variables, while independent sample t-tests and ANOVA were applied to compare quantitative study variables with two or more categories, respectively. All statistical analyses were performed using Microsoft Excel (Microsoft Corporation, Redmond, WA, USA) and IBM SPSS Statistics for Windows, version 25.0 (released 2017; IBM Corp., Armonk, NY, USA). A p-value < 0.05 was considered statistically significant. Logistic regression was applied to identify factors influencing binary outcome variables, with results presented as ORs with 95% CIs and corresponding p-values.

## Results

Among the 150 patients included in this study, the majority were from the age group 65 to 70 years (n = 97, 64.7%). Only six patients were above 80 years of age. Sixty percent of patients were male. The majority of the study participants were from middle and lower socioeconomic strata (n = 81 and 58, respectively; 54% and 37%). The most common diagnosis was head and neck malignancy, followed by gastrointestinal tract and breast cancer. Seventy-five patients (50%) had locally advanced carcinoma. Patients detected to have distant metastasis on screening were excluded from the study. Chemotherapy indications included adjuvant, neoadjuvant, and radical chemotherapy. Clinicopathological characteristics are summarized in Table 1.

**Table 1:** Clinicopathological characteristics of patients CT, chemotherapy; CTRT, concurrent chemoradiation therapy; GIT, gastrointestinal tract; GUT, genitourinary tract; HNC, head and neck cancer; RT, radiation therapy; SES, socioeconomic strata

Clinical characteristics	n (%)
Age	
65-70	97 (64.7)
71-75	39 (26)
76-80	8 (5.3)
>80	6 (4)
Gender	
Male	90 (60)
Female	60 (40)
SES [8]	
Upper	11 (7.3)
Middle	81 (54)
Lower	58 (38.7)
Diagnosis	
HNC	73 (48.7)
GIT	32 (21.3)
Breast	22 (14.7)
Gynecological	14 (9.3)
Lung	7 (4.7)
GUT	2 (1.3)
Stage	
I	5 (3.33)
II	22 (14.67)
III	48 (32)
IVA	68 (45.33)
IVB	7 (4.67)
Indication of chemotherapy	
Adjuvant CT	28 (18.67)
Adjuvant CTRT	28 (18.67)
Radical CTRT	28 (18.67)
Neoadjuvant CT	16 (10.67)
Neoadjuvant CTRT	1 (0.67)
Neoadjuvant → radical CTRT	43 (28.67)
Neoadjuvant CTRT → adjuvant CT	6 (4)

GA was performed at baseline for all patients and included evaluation of functionality, nutrition, comorbidities, psychological and social well-being, and medication reconciliation for polypharmacy, as summarized in Table 2.

**Table 2:** GA at baseline and distribution over CTCAE grade * Wald statistic (p-value) and OR (95% CI) were computed for the event “severe CTCAE group.” p < 0.05 was considered statistically significant. CCI, Charlson Comorbidity Index; CTCAE, Common Terminology Criteria for Adverse Events; ECOG PS, Eastern Cooperative Oncology Group Performance Status; GA, geriatric assessment; GDS-4, Geriatric Depression Scale-4; MOS-SSS, Medical Outcomes Study Social Support Survey; TUG, Timed Up and Go

GA parameter	Category	Total n (%)	CTCAE max mild	CTCAE max severe	Wald test statistic (p-value)*	OR (95% CI)*
ECOG PS [7]	1	48 (32)	33	15	0.064 (0.800)	Ref
	2	102 (68)	68	34		1.10 (0.527-2.297)
BMI [9]	≤18.50	40 (26.67)	26	14	0.135 (0.713)	Ref
	>18.50	110 (73.33)	75	35		0.867 (0.404-1.806)
TUG [13]	≤20	125 (83.33)	84	41	0.006 (0.938)	Ref
	>20	25 (16.67)	17	8		0.964 (0.384-2.418)
History of falls	No	115 (76.67)	23	12	0.054 (0.816)	Ref
	Yes	35 (23.33)	78	37		1.10 (0.494-2.448)
CCI [14]	≤2	78 (52)	56	22	1.463 (0.225)	Ref
	>2	72 (48)	45	27		1.527 (0.769-3.033)
GDS-4 [10]	Normal	91 (60.67)	60	31	0.366 (0.545)	Ref
	Suggestive	58 (38.67)	41	17		0.803 (0.394-1.636)
	Almost confirmed	1 (0.67)	0	1		-
MOS-SSS [11]	≤80	75 (50)	51	24	0.03 (0.862)	Ref
	>80	75 (50)	50	25		1.062 (0.537-2.103)
Polypharmacy	No	50 (33.33)	36	14	0.74 (0.389)	Ref
	Yes	100 (66.67)	65	35		1.385 (0.660-2.907)

Patients received chemotherapy according to institutional protocol following multidisciplinary team discussion. They received single-agent or combination chemotherapy based on their disease indication. Chemotherapy agents included platinum compounds (cisplatin, carboplatin, and oxaliplatin), taxanes (paclitaxel and docetaxel), fluoropyrimidines (5-FU and capecitabine), anthracyclines (doxorubicin), etoposide, gemcitabine, and methotrexate. Supportive care medications were administered as needed, including prophylactic growth factor (granulocyte colony-stimulating factor) support according to institutional policy.

Of 150 patients, 72 (48%) completed all planned cycles without dose reduction or delay. Thirteen patients (8.7%) required a cycle delay, 42 (28%) required a dose reduction, and 23 (15.3%) required both.

The most common chemotherapy-related ADRs were nausea, vomiting, anorexia, diarrhea, oral mucositis, and hematologic toxicities, as detailed in Table 3. A total of 101 patients reported mild-grade ADRs (CTCAE grades 0, 1, and 2), and 49 patients reported severe-grade ADRs (CTCAE grades 3, 4, and 5), representing 67.33% and 32.66% of patients, respectively. A total of nine deaths (6%) occurred during chemotherapy, corresponding to CTCAE grade 5.

**Table 3:** Chemotherapy ADRs among the study population CTCAE was used to grade the severity of adverse drug reactions [12]. ADR, adverse drug reaction; CTCAE, Common Terminology Criteria for Adverse Events

ADR	Grade 0	Grade 1	Grade 2	Grade 3	Grade 4	Grade 5
Anorexia	10	73	65	2	0	9
Nausea	46	72	30	2	0	
Vomiting	58	36	45	11	0	
Diarrhea	113	15	16	5	1	
Constipation	53	75	22	0	0	
Mucositis	75	18	47	10	0	
Peripheral neuropathy	116	21	10	3	1	
Anemia	118	26	1	5	1	
Neutropenia	138	6	2	3	1	
Thrombocytopenia	141	4	4	1	0	
Renal dysfunction	139	9	1	0	0	
Rash	85	27	35	3	0	
Fatigue	6	69	72	3	0	
Pain	12	41	94	3	0	

Chi-square analysis showed that baseline GA parameters were not associated with CTCAE grade (p > 0.05, not significant), as detailed in Table 2.

**Figure 1:**
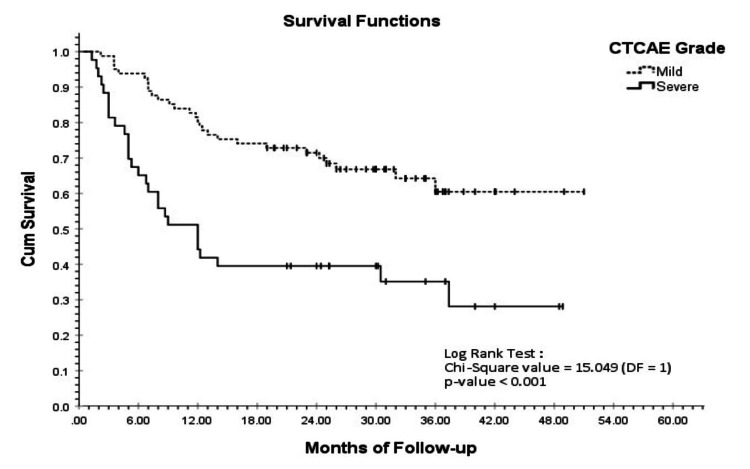
KM survival curve for CTCAE grades: mild vs. severe CTCAE, Common Terminology Criteria for Adverse Events; KM, Kaplan-Meier

KM survival analysis at the end of four years of follow-up revealed that the average estimated survival for patients with mild-grade ADRs was 36.9 months, with a cumulative survival probability of 0.605 (SE = 0.065), whereas the average estimated survival for patients with severe-grade ADRs was 21.5 months, with a cumulative survival probability of 0.281 (SE = 0.085). This difference was statistically significant (log-rank test p < 0.001) at the 5% level of significance (Figure 1).

**Figure 2:**
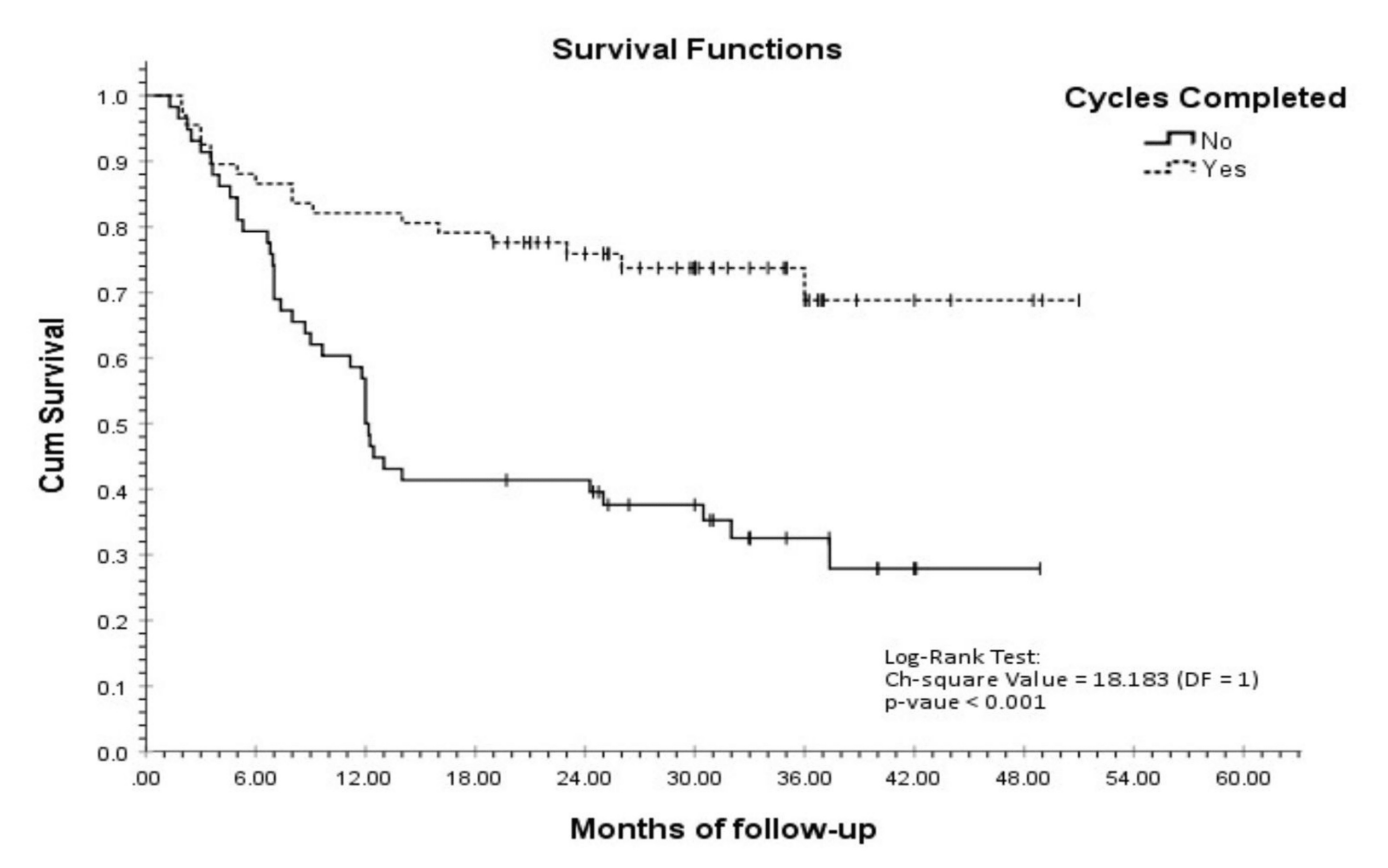
KM survival curve for chemotherapy cycles: completed vs. not completed KM, Kaplan-Meier

Further KM analysis at the end of four years showed that patients who completed their chemotherapy cycles had an average estimated survival of 39.4 months, with a cumulative survival probability of 0.688 (SE = 0.070), while patients who did not complete chemotherapy cycles had an average estimated survival of 22.3 months, with a cumulative survival probability of 0.279 (SE = 0.070). This difference was also statistically significant (log-rank test p < 0.001) at the 5% level of significance (Figure 2).

## Discussion

The global burden of cancer is rapidly increasing, particularly among older adults. A report from the National Cancer Registry Programme, India, indicates that nearly 40-45% of all cancers occur in individuals aged ≥60 years [15]. Similarly, a multicentric observational study conducted across 17 institutes in India reported that 36% of patients (n = 97,962) were aged ≥60 years. In this study, the median age of older patients with cancer was 67 years (IQR 63-72), and head and neck malignancies were the most prevalent, accounting for 11,158 cases (19.6%) [16].

Our study evaluated geriatric cancer patients (≥65 years), with a median age of 69 years, and the majority of patients were between 65 and 75 years of age (90.7%, n = 136). Only patients treated with curative intent were included; those with metastatic disease receiving palliative chemotherapy were excluded. The higher proportion of relatively younger patients reflects the more palliative approach toward older patients in the region. In our cohort, 48.7% of patients (n = 73) were diagnosed with squamous cell carcinoma of the head and neck, and 75 patients (50%) had clinically locally advanced disease. Most of these patients had a history of tobacco chewing (≥90%), a common practice in this geographical area. The study population predominantly came from rural backgrounds, and due to limited awareness and access to healthcare, many patients presented with locally advanced or metastatic disease.

In a multi-institutional study of 500 patients, Hurria et al. reported grade 3-5 toxicity in 53% of patients (39% grade 3, 12% grade 4, and 2% grade 5). Using this dataset, a predictive model for grade 3-5 toxicity was developed, incorporating GA variables, laboratory test values, and patient, tumor, and treatment characteristics [17]. In our study population of 150 patients, 49 (32.67%) developed severe ADRs (CTCAE grade 3-5). Baseline GA included functional, nutritional, psychological, and social domains. Comorbidities and polypharmacy were present in 72 (48%) and 100 (66.67%) patients, respectively. A population-based study by Jorgensen et al. reported that 35% of cancer patients aged ≥70 years used five or more medications at diagnosis prior to receiving anticancer treatment [18]. Polypharmacy increases the risk of drug interactions, enhancing toxicities, and potentially leading to hazardous health outcomes.

CGA has emerged as an important tool for predicting chemotherapy-related toxicity and treatment tolerance in older patients, facilitating individualized treatment decisions [19]. In our cohort, 96% of patients had deficits in one or more GA components; however, association analysis did not show a statistically significant correlation with severe ADRs.

Geriatric cancer patients often empirically receive chemotherapy at reduced dose intensity (RDI). In the McKesson Specialty Health/US Oncology iKnowMed electronic health record database, Denduluri et al. analyzed community oncology practices in the United States and found that RDI was more common in older, obese, and functionally restricted patients [20]. Similarly, Bouleftour et al. reported that in 179 patients over 70 years old receiving first-course chemotherapy for solid tumors, 69.8% received standard doses, while 30.2% experienced ADRs. Toxicities occurred in 83.2% of standard-dose patients versus 68% of ADR patients [21]. In our cohort, 42 patients (28%) required dose reduction, 13 (8.7%) required cycle delays, and 23 (15.3%) required both.

The rate of completion of planned chemotherapy cycles may serve as a surrogate marker of survival outcomes, integrating determinants such as treatment tolerance, toxicity burden, and maintenance of dose intensity. Wildes et al. found that in older adults, curative intent therapy was significantly associated with therapy completion (OR 4.97 (95% CI 1.21-18.81)) and lower mortality (HR 0.15 (0.06-0.42)) [22]. In our cohort, 72 patients (48%) completed all planned cycles. KM survival analysis revealed significantly higher average estimated survival for patients completing all planned chemotherapy cycles compared to those who did not (39.4 months vs. 22.3 months; log-rank p < 0.001). This underscores the importance of completing planned chemotherapy cycles in geriatric patients treated with curative intent.

Higher-grade toxicities reflect reduced physiological reserve, impaired treatment tolerance, and the need for dose reductions, treatment delays, or early discontinuation, ultimately compromising intended dose intensity [23]. In our cohort, KM survival analysis showed average estimated survival of 36.9 months for mild-grade CTCAE versus 21.5 months for severe-grade CTCAE, a statistically significant difference (log-rank p < 0.001). Future research is needed to identify interventions that reduce high-grade toxicities, improve chemotherapy completion rates, and enhance survival in geriatric cancer patients.

Strengths and limitations

The study presents both strengths and limitations. A major strength lies in its distinction as one of the few prospective studies from Western India addressing the unmet need for geriatric-specific chemotherapy toxicity data. GA was performed for each patient using standardized, validated assessment tools, and the study included a clearly defined geriatric cancer population with systematic data collection and follow-up.

There are several limitations to the present study. It is a single-center, observational study. The study included patients with several tumor types and multiple chemotherapy drugs, and due to the modest sample size, subgroup analysis could not be performed. Head and neck cancers are the most common malignancy in the geographical area where this study was conducted. The majority of patients had locally advanced head and neck cancer, which may impact GA parameters, chemotherapy ADRs, and survival outcomes. GA was performed only at baseline, so it was not possible to determine the directionality of the relationships observed. The reasons for ADRs were not captured, and the association between ADR and its impact on treatment efficacy cannot be addressed with this dataset. These limitations restrict the generalizability of the results, and caution should be taken when extrapolating these findings to different cultural or societal contexts. Longitudinal, multicentric cohort studies are needed to confirm the findings from this study.

## Conclusions

Due to population aging and the increased prevalence of cancer with age, the global burden of geriatric cancer is rising. These patients often present with frailty, comorbidities, polypharmacy, and a higher incidence of severe ADRs to standard-dose chemotherapy. Dose modifications and treatment delays are frequent. This study highlights the importance of completing planned chemotherapy cycles in geriatric cancer patients treated with curative intent. Severe cancer treatment-related toxicities negatively impact patient outcomes, emphasizing the need for personalized toxicity management.
